# Neuroprotection by Argon Ventilation after Perinatal Asphyxia: A Safety Study in Newborn Piglets

**DOI:** 10.1371/journal.pone.0113575

**Published:** 2014-12-02

**Authors:** Thomas Alderliesten, Laurent M. A. Favie, Robert W. Neijzen, Volker Auwärter, Cora H. A. Nijboer, Roland E. J. Marges, Carin M. A. Rademaker, Jürgen Kempf, Frank van Bel, Floris Groenendaal

**Affiliations:** 1 Department of Neonatology, Wilhelmina Children's Hospital/University Medical Center Utrecht, Utrecht, The Netherlands; 2 Department of Clinical Pharmacy, Division of Laboratory Medicine and Pharmacy, University Medical Center Utrecht, Utrecht, The Netherlands; 3 Department of Forensic Toxicology, Institute of Forensic Medicine, University Medical Center Freiburg, Freiburg, Germany; 4 Laboratory of Neuroimmunology and Developmental Origins of Disease, University Medical Center Utrecht, Utrecht, The Netherlands; 5 Department of Medical Technology, University Medical Center Utrecht, Utrecht, the Netherlands; Hôpital Robert Debré, France

## Abstract

Hypothermia is ineffective in 45% of neonates with hypoxic-ischemic encephalopathy. Xenon has additive neuroprotective properties, but is expensive, and its application complicated. Argon gas is cheaper, easier to apply, and also has neuroprotective properties in experimental settings. The aim was to explore the safety of argon ventilation in newborn piglets.

**Methods:**

Eight newborn piglets (weight 1.4–3.0 kg) were used. Heart rate, blood pressure, regional cerebral saturation, and electrocortical brain activity were measured continuously. All experiments had a 30 min. baseline period, followed by three 60 min. periods of argon ventilation alternated with 30 min argon washout periods. Two animals were ventilated with increasing concentrations of argon (1h 30%, 1 h 50%, and 1 h 80%), two were subjected to 60 min. hypoxia (FiO_2_ 0.08) before commencing 50% argon ventilation, and two animals received hypothermia following hypoxia as well as 50% argon ventilation. Two animals served as home cage controls and were terminated immediately.

**Results:**

Argon ventilation did not result in a significant change of heart rate (mean ± s.d. −3.5±3.6 bpm), blood pressure (−0.60±1.11 mmHg), cerebral oxygen saturation (0.3±0.9%), electrocortical brain activity (−0.4±0.7 µV), or blood gas values. Argon ventilation resulted in elevated argon concentrations compared to the home cage controls (34.5, 25.4, and 22.4 vs. 7.3 µl/ml).

**Conclusion:**

Ventilation with up to 80% argon during normoxia, and 50% argon after hypoxia did not affect heart rate, blood pressure, cerebral saturation and electrocortical brain activity. Clinical safety studies of argon ventilation in humans seem justified.

## Introduction

Perinatal asphyxia in full term neonates is an important cause of mortality and morbidity. Moderate hypothermia is the standard neuroprotective treatment, but is still ineffective in 45% of cases. [1] This underlines the need for additional neuroprotective interventions. [2]

Brain injury following hypoxia-ischemia is partly mediated by activation of the N-methyl-D-aspartate (NMDA) subtype of the glutamate receptor. [3] The noble gas xenon exhibits non-competitive antagonism of this NDMA receptor and results of its use as an additive neuroprotective agent are promising. [4]–[6] However, Xenon is an expensive gas and therefore requires complex closed-circuit ventilation systems. [7], [8] This makes large scale clinical use of Xenon less feasible.

Recently, argon gas has been shown to have neuroprotective properties in cell cultures of dissociated neurons, in organotypic hippocampal slice cultures, and in vivo models. [9]–[14] In contrast with xenon, argon is a more abundant and cheaper noble gas. It can be obtained as a pure gas of pharmaceutical quality at low costs and therefore does not require complex ventilator setups.

At present data on the safety of argon ventilation in larger animals is scarce. Therefore, the aim of the present study was to explore the safety of argon ventilation in newborn piglets.

## Materials and Methods

### Animal preparation and instrumentation

The experimental protocol was approved by the Animal Care Committee of Utrecht University, The Netherlands. Eight newborn Dutch store piglets with a mean age of 4 days (range 2–7 days) and mean body weight of 2275 g (range 1400–3000 g) were used. The animals were sedated using a intramuscular injection consisting of midazolam (0.7 mg/kg), ketamine (13 mg/kg), and atropine (0.02 mg/kg). Thiopental (4 mg/kg) was administered before endotracheal intubation.

An intravenous catheter was inserted in an ear vein for continuous infusion of glucose 10% and sedatives. General anesthesia was maintained throughout the experiment with midazolam (2 mg/kg/h) and morphine (40 µg/kg/h). Pancuronium bromide (1 mg/kg/h) was used for muscle paralysis.

For monitoring mean arterial blood pressure (MABP) and sampling of arterial blood gasses, a catheter was surgically inserted in the right femoral artery. The blood was heparinized with 2.5 U/ml (2 ml/h). The total fluid administration rate was 5 ml/kg/h.

Rectal temperature was maintained between 38.5 and 39.5 by using a heated table top matrass and a forced-air warming system (Bair Hugger, Arizant Healthcare Inc., Eden Prairie, MN).

### Argon ventilation

**Figure 1:**
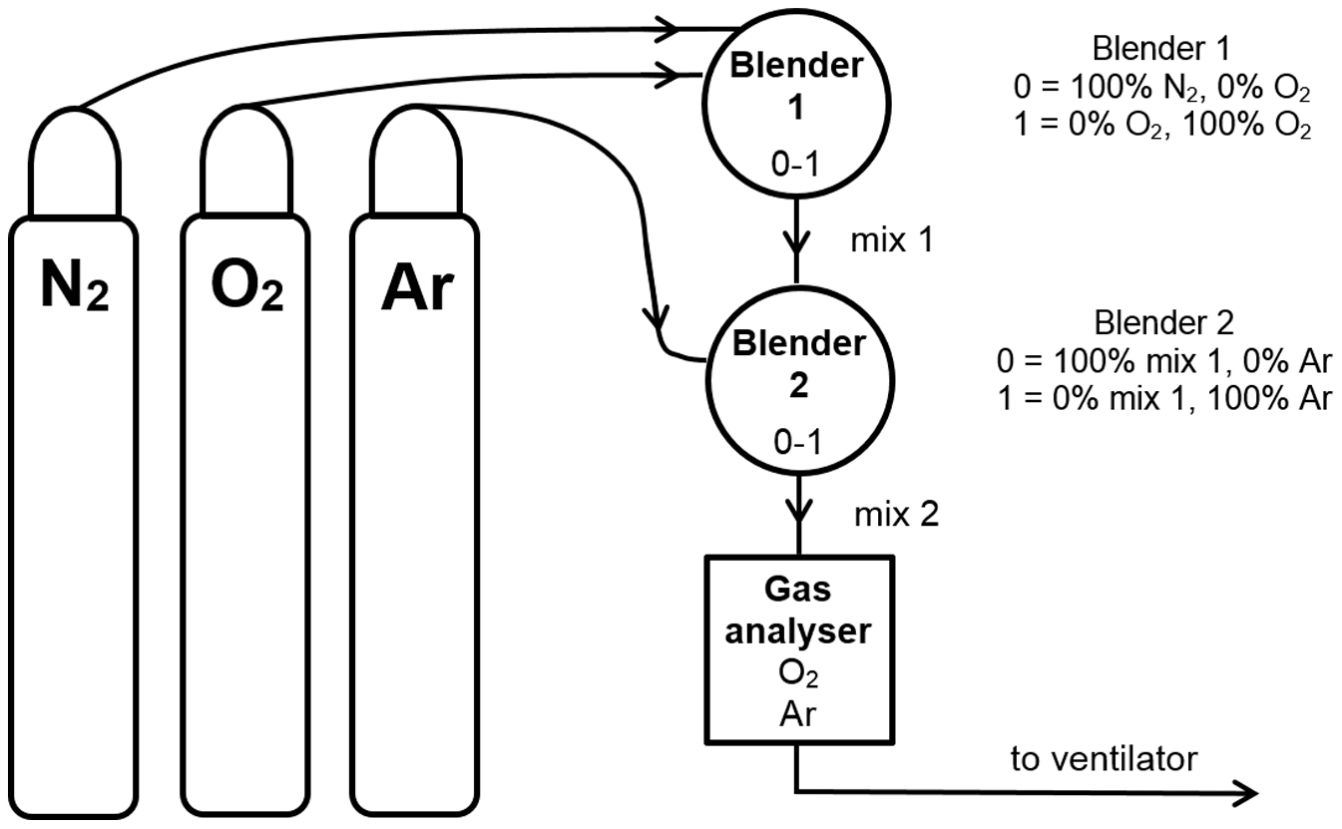
Schematic of ventilator setup.

Medical grade argon, with a purity of > 99.995% was used during the animal experiments (Linde Gas Therapeutics Benelux BV, The Netherlands). The piglets were mechanically ventilated by using a continuous flow, pressure-controlled ventilator (Stephanie, Fritz Stephan GmbH, Gackenbach, Germany). The ventilator was technically adapted to enable ventilation with oxygen, argon, and nitrogen. The three gases were mixed by using two gas blenders. Mixture 1, containing oxygen and nitrogen, was mixed in the first blender. In the second blender, mixture 1 was blended with argon to obtain the final mixture (mixture 2). Figure 1 displays the setup for argon ventilation.

The final gas mixture was analyzed by a gas analyzer (KG6050 Dual gas analyzer, Hitech Instruments Ltd, Luton, Bedfordshire, United Kingdom) to quantify oxygen and argon quantity in the mixture. The gas analyzer has a resolution of 0.1%, and an accuracy of ±1.0% for oxygen and argon. These adjustments enabled gas concentrations of 0–100% for all three gasses (i.e. oxygen, nitrogen, and argon).

### Parameters for safety measurements

Heart rate (HR), MABP, arterial SO_2_, end-tidal CO_2_, and rectal temperature were monitored using a M1094B patient monitor (Philips, Best, The Netherlands). These physiological parameters were recorded and stored at 1 Hz on a personal computer for off-line analysis (Poly 5, Inspector Research Systems, Amsterdam, The Netherlands).

An Olympic 6000 (Natus Medical Systems, Seattle, WA) cerebral function monitor was used to monitor electrocortical brain activity throughout the experiments. This monitor continuously records the amplitude-integrated electroencephalogram (aEEG), real time raw EEG, and electrode impedance at 100 Hz. The (a)EEG signal was obtained from a pair of needle electrodes spaced 1.5 cm apart placed on the left side of the scalp with a central (frontal) reference electrode. [15], [16]

The regional cerebral saturation (rScO_2_) was monitored by using a two wavelength (730 and 810 nm) Near-InfraRed Spectrometer (INVOS 4100, Covidien, Mansfield, MA) with a transducer containing a light emitting diode and two distant sensors (i.e. at 30 and 40 mm).

The transducer was placed in a cross-cerebral configuration on shaved skin just between the eyes and ears. The sensor was fixated by using an elastic bandage and was carefully positioned to avoid contact with the aEEG electrodes.

Arterial pH, base excess, arterial pO_2_ and pCO_2_, glucose and lactate concentration were obtained by blood gas analysis. A blood sample was drawn during baseline and subsequently after each change in ventilation settings.

### The Experimental Protocol

Two animals served as home cage controls for histopathologic examination (see below) and where therefore terminated using pentobarbital (100 mg/kg) upon arrival at the lab. In the remaining 6 animals the experiment contained a 30 min. baseline period after intubation as a washout of the drugs used for induction. Subsequently 3 periods of 60 min. argon ventilation were alternated by 30 min of normoxic ventilation without argon to allow for argon washout.

**Figure 2:**
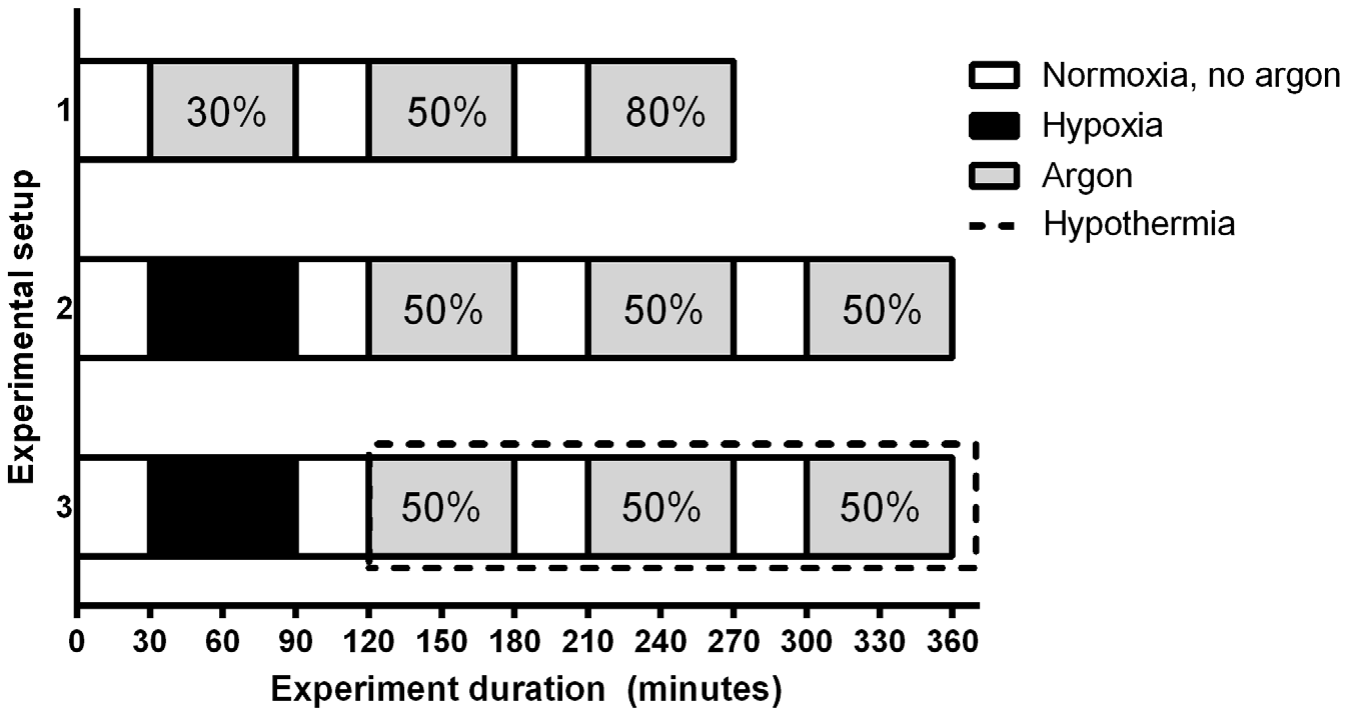
Schematic representation of the three steps of the experimental setup.

Figure 2 is a schematic representation of the three experimental setups that were used to explore the effect of argon ventilation in different clinical settings:

1. *Normoxia.* The first two animals were used to examine the effects of increasing concentrations of argon (i.e. 30%, 50%, and 80%) in normoxic, normothermic animals.
2. *Hypoxia*. After the baseline period of 30 min, two additional animals were subjected to hypoxia by decreasing the FiO_2_ to 0.08 for 60 min, after which 50% argon ventilation was started to study the effects of argon ventilation after hypoxia.
3. *Hypoxia + hypothermia*. The final two animals were subjected to 60 min of hypoxia, followed by therapeutic hypothermia in addition to 50% argon ventilation to mimic the most likely clinical setting in which argon might be used.

In the 4 hypoxic animals 50% argon (as opposed to 80% argon) was used since this is the maximum concentration likely to be used in a clinical setting where additional oxygen may be needed for neonates with perinatal asphyxia in addition to GBS infection or meconium aspiration. During therapeutic hypothermia, the rectal temperature of the two animals was maintained at 34–35°C. Hypothermia was achieved by using small packs of refrigerated saline. Animals were terminated after the third period of argon ventilation with an overdose pentobarbital (100 mg/kg i.v.).

### Histopathology

Termination of the animals was directly followed by transcardial perfusion with 4% paraformaldehyde in phosphate buffered salt. Brains were post-fixed in 4% formaline for 5–7 days, dehydrated (30–100% ethanol), and subsequently embedded in paraffin. Coronal sections of 4 µm were cut at hippocampal level.

We studied neuronal damage in detail by assessing pyknotic nuclei in HE-stained sections as a marker of dying neurons and loss of MAP2 staining as a specific marker of neuronal integrity. [17], [18] These markers might be signs of argon toxicity.

Deparaffinized sections were stained with hematoxylin-eosin (HE; Klinipath, Duiven, the Netherlands) or were incubated with mouse-anti-MAP2 (microtubule associated protein 2) antibody (Exbio, Vestec, Czech Republic) followed by biotin-labeled horse-anti-mouse antibody (Vector Laboratories, Burliname, CA). Visualization was performed by using Vectastain ABC kit (Vector Laboratories) and diaminobenzidine (Sigma-Aldrich, Steinheim, Germany). Photographs were made using a Zeiss Axio Lab A1 microscope and Icc5 camera and analyzed using ZEN2012 software (Carl Zeiss, Oberkochen, Germany).

### Argon analysis

To assess the concentration of argon that reached the blood, two 1 ml blood samples per animal were drawn during the final minutes of argon ventilation, just before termination. In addition, blood samples were also drawn from the control animals before termination. These samples were immediately transferred into headspace vials, sealed airtight, and frozen at -20°C until analysis. Two air samples were also taken to determine the argon concentration in lab air. Calibration samples were prepared in the University Medical Center Freiburg, Germany by adding 10, 20, 30, 40, 50, 60, 70, 80, 90 and 100 µL of argon (Air Liquide Deutschland GmbH, Düsseldorf, Germany; purity > 99.999%,) to blank piglet blood using a gas tight syringe. Prior to analysis, animal samples and calibration samples were stored at room temperature for 2.5 h to reach equilibrium. After equilibrium, argon (Ar, m/z 40) was measured using nitrogen (N_2,_ m/z 28) as an internal standard. Ar/N_2_ ratios were plotted against added argon concentration (µl/ml).

Analysis was performed using an Agilent 6890N gas chromatography system combined with a Agilent 5973 Series mass selective detector (Agilent, Waldbronn, Germany) and a PAL headspace autosampler (CTC Analytics AG, Zwingen, Switzerland). The samples were incubated at 30°C for 30 seconds and 250 µL gas from the headspace was injected into the gas chromatography – mass spectrometry (GC-MS) system (split injection mode 500∶1).

Separation was performed on an PoraPLOT Q column (25 m×0.25 mm) (Agilent, Waldbronn, Germany) using the following temperature gradient: 40°C for 3 min, increased to 210°C at 50°C/min and holding 210°C for 2.1 min. Helium 4.6 was used as carrier gas at a constant flow rate of 0.8 mL/min. The MS conditions were as follows: transfer line heater 280°C; ion source temperature 230°C; quadrupole temperature 150°C; electron impact ionization mode, and ionization energy 70 eV. Analysis was performed in full scan mode analyzing a mass range from 10 to 70 amu with a scan time of 0.2 seconds.

### Data processing and analysis

The three periods of argon ventilation yielded 5 switches per animal (figure 2) and thereby 30 switches (i.e. 6 animals with argon, 5 switches per animal) in total. Data was used from representative segments of at least 15 min. that were selected at the beginning and at end of each argon or washout period. The change in physiological parameters was always calculated by subtracting the segment with argon ventilation from the segment without argon ventilation.

All physiological data were imported in SignalBase (v7.7.8, UMC Utrecht, The Netherlands) and analyzed together.

### Statistics

The 5 argon-normoxia switches per animal and use of 6 animals (30 transitions) enabled us to detect a change of more than 10% in any parameter, which was considered clinically relevant, with a power of 80%. Statistical analysis was performed by using a mixed models approach in R (version 2.14.1; http://www.r-project.org) with the *nlme* package. The different physiological parameters were used as the dependent variable, the individual piglet was used as a random factor, and argon ventilation (y/n) during an epoch of recorded data, hypoxia in the experimental setup (y/n), and hypothermia in the experimental setup (y/n) were investigated as fixed factors. A p-value <0.05 was considered statistically significant.

## Results

### Concentrations in blood

With the calibration samples a calibration line was made: Ar/N_2_ ratio  = 0.0003*blood concentration (µl/ml) +0.0583, with a R^2^ of 0.9949.

**Figure 3:**
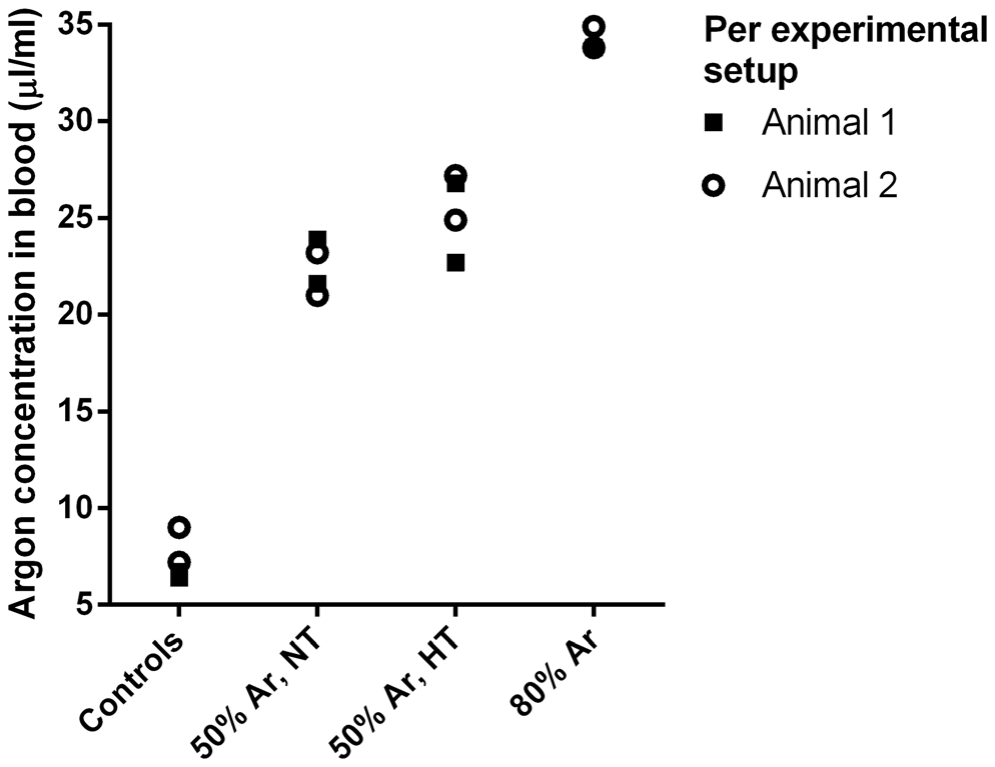
Concentrations of argon gas (µl/ml) in blood in home cage controls, animals ventilated under normoxic conditions, animals ventilated with argon after hypoxia, and animals ventilated with argon after hypoxia that subsequently underwent hypothermia. Separate animals within one experimental setup are displayed with different symbols (open circles and closed squares).

Results of the argon analysis in blood are demonstrated in figure 3. Argon concentrations were higher in piglets that were ventilated with 50% argon as compared to the control piglets (med. 23.6 µl/ml vs. 7.0 µl/ml) The value of the control piglets is extrapolated, and below the lowest value of the calibration line. In addition, there was a difference in argon concentrations between piglets that were ventilated with 80% argon as compared to piglets that were ventilated with 50% argon upon termination (med. 34.5 µl/ml vs. 23.6 µl/ml). Analysis of laboratory air did not show altered argon background concentrations at the time of the experiment (data not shown).

### Physiology

**Figure 4:**
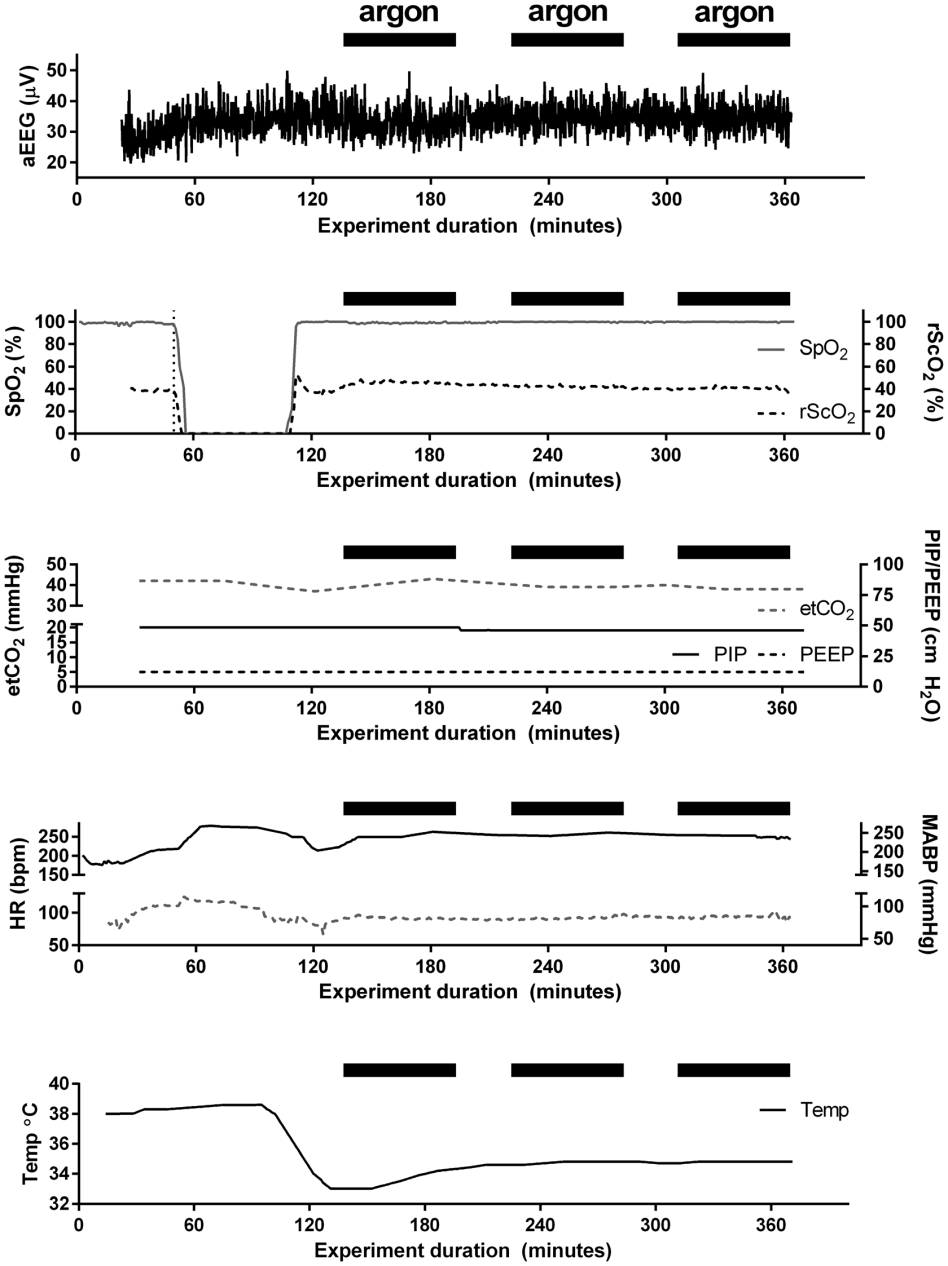
Recorded physiological data from representative animal receiving therapeutic hypothermia. Black horizontal bars represent periods of argon ventilation.

Figure 4 displays representative recordings of physiological parameters of an animal that underwent hypoxia and subsequent hypothermia. During hypoxia HR and MABP increased, and rScO_2_ decreased, whereas aEEG remained stable during the one hour hypoxia. In the hypoxic animals, hypoxia resulted in metabolic acidosis (pH 7.03±0.09, base excess −19.9±3.0 mmol/l, lactate 11.8±1.5 mmol/l). These values normalized during re-oxygenation.

**Figure 5:**
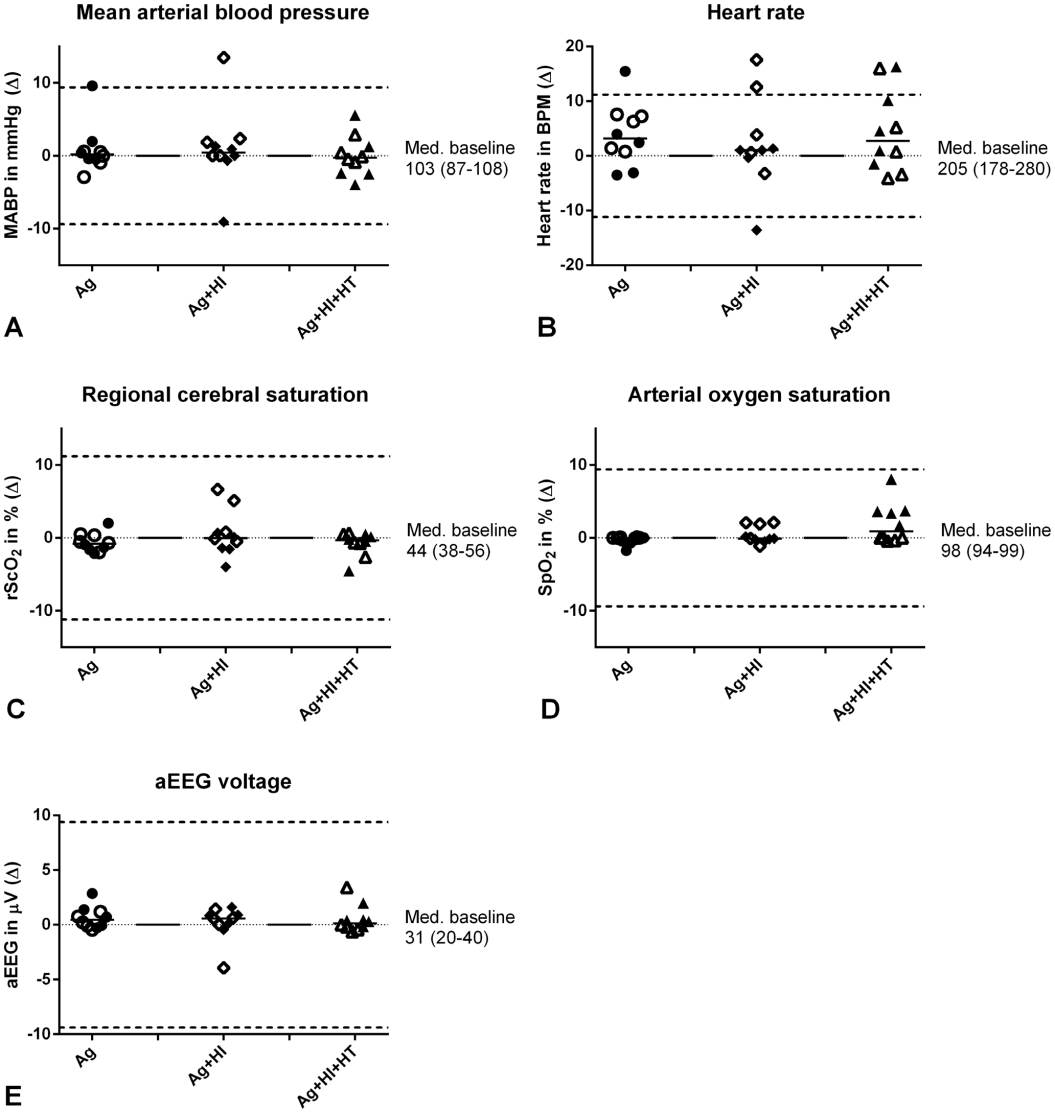
Calculated differences in monitored parameters (normoventilation – argon ventilation) displayed as absolute difference. For reference, the median value of the 6 animals during baseline is added to the right of each panel. Each dot represents a single switch from argon to normoventilation or vice versa. Ag: argon, HI: hypoxia-ischemia, HT: hypothermia.

In the mixed model analysis, ventilation with argon did not result in a significant change of HR, mean arterial blood pressure, rScO_2_ or aEEG (see table 1). Having received hypoxia, and undergoing hypothermia did not have significant effects on HR, aEEG, and rScO_2_, but hypothermia did cause a decrease in MABP. Figure 5 displays the absolute changes in physiological parameters induced by switching between argon ventilation and normoxic ventilation without argon, showing that the median change is always very close to zero.

**Table 1:** Coefficients of argon, hypoxia and hypothermia after multivariable analysis of mean arterial blood pressure (MABP), heart rate, aEEG voltage and regional cerebral saturation (rScO_2_).

	MABP (mmHg, 95% CI)	Heart rate (bpm, 95% CI)	aEEG voltage (µV, 95% CI)	rScO_2_ (%, 95% CI)
Intercept	98.5	195	31	43.3
Argon	−0.6 (−2.8–1.6)	3 (−10–4)	−0.5 (−2–1)	0.3 (−1.5–2.0)
Post-hypoxia	−7.9 (−28.3–12.5)	35 (−31–101)	1 (−10–12)	8.9 (−9.1–26.9)
Hypothermia	−24.2 (−44.6–−3.8)	−30 (−96–36)	1 (−10–12)	−6.7 (−24.6–11.3)

### Histology

**Figure 6:**
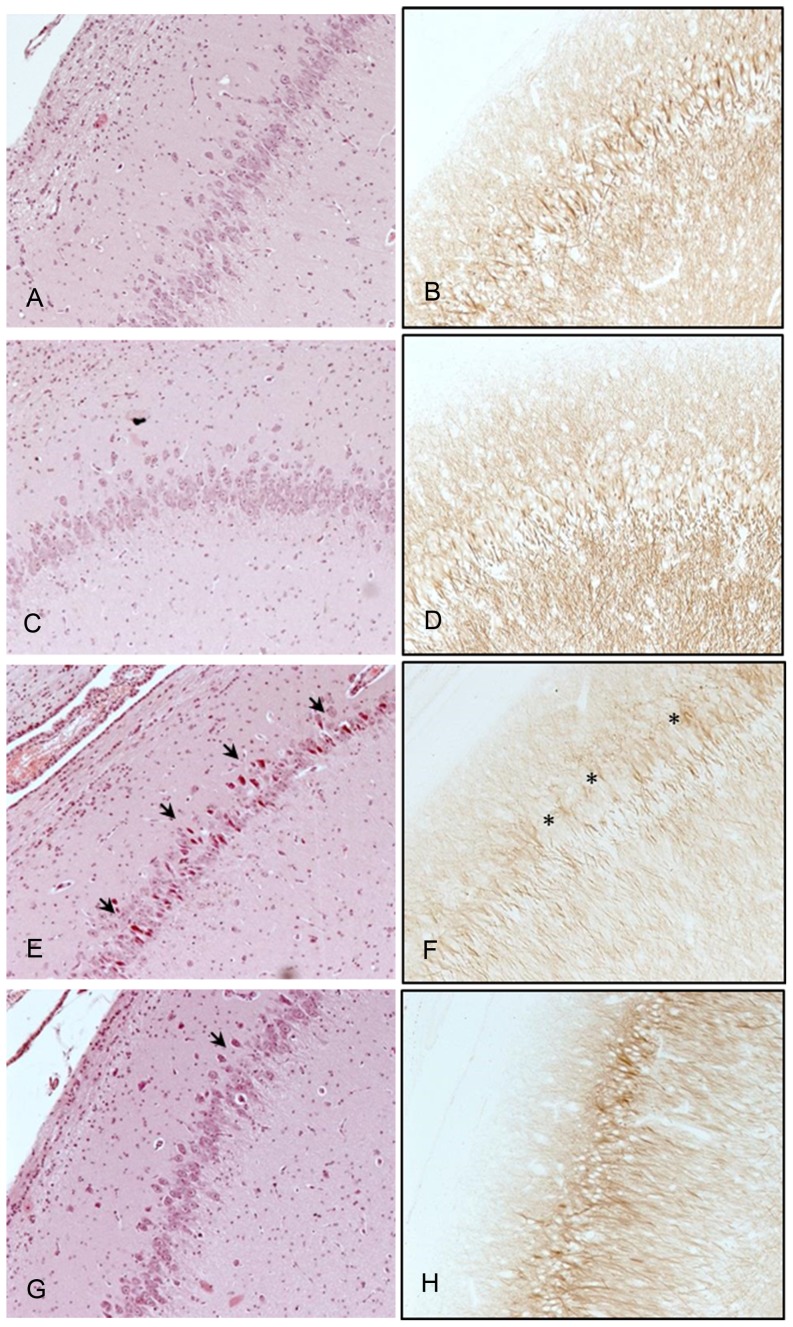
Hematoxylin-eosin staining (left) and microtubule associated protein 2 (right) stained slides of the hippocampus of (top-to bottom): A,B: home cage controls, C,D: animals ventilated with argon under normoxic conditions, E,F: animals ventilated with argon after hypoxia, and G,H: animals ventilated with argon after hypoxia that subsequently underwent hypothermia. No differences between home cage controls and argon ventilated normoxic animals were demonstrated. Marked brain damage was seen in hypoxic animals demonstrated by pyknotic nuclei (arrowheads in panels E and G) and MAP2 loss (asterisks in panel F).

In histopathologic examination, HE staining of the hippocampus did not demonstrate an increase in pyknotic cell nuclei in piglets that were ventilated with argon under normoxic conditions as compared to the control piglets (Figure 6, panels A vs. C). The amount of MAP2 staining in the hippocampus was also comparable between the control piglets and piglets ventilated under normoxic conditions (Figure 6, panels B vs. D).

Figure 6 also presents HE and MAP2 stained hippocampal slides of piglets that underwent hypoxia before argon ventilation. Brain injury could be demonstrated in HE slides by pyknotic cell nuclei in multiple areas of the hippocampus (Figure 6, panels E and G). In addition, there was a loss in MAP2 most markedly seen in piglets undergoing hypoxia without subsequently receiving hypothermia. (Figure 6, panel F). Although less severe, HE and MAP2 stained slides of the cerebral cortex had an aspect similar to the hippocampus, with pyknotic cell nuclei and MAP2 loss (data not shown).

## Discussion

In the present study, the potential effect of argon ventilation on important physiological parameters was explored. A subgroup of animals was subjected to hypoxia and subsequent hypothermia to mimic the conditions during which argon will be used in clinical settings. Being exposed to hypoxia, and undergoing hypothermia tended to show some effects on HR and blood pressure. However, argon ventilation did not have any effect on these parameters. In addition, argon did not have negative effects on important cerebral functions such as aEEG or cerebral saturation, and did not seem to induce additional brain injury as assessed by histopathology in normoxic animals.

Our results show a dose-dependent systemic exposure for argon and that this exposure can be quantified in blood samples. Furthermore, increased blood argon concentrations following argon ventilation up to 80% in normoxic animals, and of 50% in hypoxic animals did not have any effect on important physiological parameters as mentioned above. In hypoxic animals the dose of 50% argon was used since this is the concentration most likely to be used in a clinical setting where additional oxygen may be needed for neonates with pulmonary problems in addition to perinatal asphyxia.

Previous studies have focused on the noble gas xenon for additive neuroprotection. Xenon demonstrated stable hemodynamics, independent of induced hypothermia. [19] Moreover, xenon ventilation up to 18 hours has been shown to be feasible in the human neonatal population in addition to hypothermia. [20] It is worth noting that xenon ventilation requires complex ventilators that recirculate the xenon gas because of the price of xenon. On top of this disadvantage, xenon has anesthetic properties at normobaric pressures that could potentially influence the neurological state of the patient, thereby complicating the assessment of the (neurological) condition of the patient by clinical examination or neuromonitoring (i.e. near-infrared spectroscopy (NIRS) or aEEG). [21]

Argon has the advantage over xenon that it is more abundant in the earth's atmosphere and therefore cheaper to produce and can be used for ventilation by only slightly modifying existing (neonatal) ventilators. Another advantage is that argon only has anesthetic properties at hyperbaric pressures and thus would not influence the neurological status of the patient in the intended clinical setting (i.e. 50% ventilation at normobaric pressures). In the past inhalation of 79% argon has been used in humans to measure coronary and myocardial blood flow [22], [23], and recently it has been used in athletes to enhance performance (see World Anti-Doping Agency: www.wada-ama.org).

The present study was not designed to assess the neuroprotective effect of argon ventilation, as we did not include a group that underwent hypoxia without subsequent argon ventilation. However, the neuroprotective effect of argon ventilation has already been shown in pure neuronal cell cultures and organotypic hippocampal slice cultures following glucose deprivation and traumatic brain injury. [9], [10] More recently, it was shown that argon significantly decreased infarction volumes and improved composite adverse outcome after unilateral internal carotid artery ligation and subsequent hypoxia in a rodent model. [12] In addition, the neuroprotective effect of argon has been demonstrated both in rodent and porcine models of cardiopulmonary resuscitation following cardiac arrest. [13], [14] In contrast to Xenon, the underlying mechanisms of neuroprotection by argon are not yet elucidated, but direct actions on specific cell death pathways in myocardial ischemia have been suggested. [24] Potential mechanisms include an increase of Bcl-2, which promotes cell survival. [12] In addition, argon modifies extracellular signal-regulated kinase 1/2 signaling in neurons and glial cells. [25] Another possible mechanism might be an indirect effect mediated by the GABA_A_ receptor. [26] Based on our observations and the previous, safe use of argon in adults in the past, as well as the efficacy studies described above, further safety studies in human neonates with perinatal asphyxia seem warranted.

Although the current study has a limited sample size, it was specifically designed to detect a 10% difference in any parameter with a power of 80%.

In conclusion, we have demonstrated that inhalation of up to 80% argon in normoxic animals and of 50% in hypoxic animals did not affect blood pressure, heart rate, cerebral saturation and electrocortical brain activity in normoxic newborn piglets, and following hypoxia with subsequent therapeutic hypothermia. We therefore suggest that clinical safety studies in human neonates seem justified.

## Supporting Information

Data S1 **The ‘Data S1’ file available on-line contains the measurements of the individual experimental animals.**
(XLSX)
