# Correction: Neuroprotection by Argon Ventilation after Perinatal Asphyxia: A Safety Study in Newborn Piglets

**DOI:** 10.1371/journal.pone.0133818

**Published:** 2015-07-22

**Authors:** Thomas Alderliesten, Laurent M. A. Favie, Robert W. Neijzen, Volker Auwärter, Cora H. A. Nijboer, Roland E. J. Marges, Carin M. A. Rademaker, Jürgen Kempf, Frank van Bel, Floris Groenendaal

The affiliations for the ninth and tenth authors are incorrect. Dr. Frank van Bel and Dr. Floris Groenendaal are not affiliated with #2 but with #1 Department of Neonatology, Wilhelmina Children's Hospital/University Medical Center Utrecht, Utrecht, The Netherlands.
